# Long-term complications after tonsil surgery: an analysis of 54,462 patients from the Swedish Quality Register for Tonsil Surgery

**DOI:** 10.3389/fsurg.2023.1304471

**Published:** 2023-12-12

**Authors:** Erik Odhagen, Fredrik Alm, Sara Axelsson, Claes Hemlin, Pia Nerfeldt, Joacim Stalfors, Ola Sunnergren

**Affiliations:** ^1^ Department of Otorhinolaryngology, Södra Älvsborgs Hospital, Borås, Sweden; ^2^ Department of Research, Education and Innovation, Södra Älvsborgs Hospital, Borås, Sweden; ^3^ Department of Otorhinolaryngology, Head and Neck Surgery, Institute of Clinical Sciences, Sahlgrenska Academy, University of Gothenburg, Gothenburg, Sweden; ^4^ School of Health Sciences, Faculty of Medicine and Health, Örebro University, Örebro, Sweden; ^5^ Department of Otorhinolaryngology, Helsingborg Hospital, Helsingborg, Sweden; ^6^ Swedish Quality Register for Tonsil Surgery, Stockholm, Sweden; ^7^ Department of Otorhinolaryngology, Karolinska University Hospital, Stockholm, Sweden; ^8^ Department of Clinical Science, Intervention and Technology (CLINTEC), Karolinska Institutet, Stockholm, Sweden; ^9^ Department of Otorhinolaryngology, Region Jönköping County, Jönköping, Sweden; ^10^ Centre for Oral Health, School of Health and Welfare, Jönköping University, Jönköping, Sweden

**Keywords:** tonsillectomy, tonsillotomy, postoperative morbidity, long-term complications, quality register research

## Abstract

**Objective:**

This study aims to evaluate long-term complications after tonsil surgery using an exploratory retrospective cohort study design based on data from the Swedish Quality Register for Tonsil Surgery (SQTS).

**Methods:**

All patients registered in the SQTS between 1 January 2009 and 31 May 2021 were eligible for the study. In this study, a long-term complication is defined as any complication persisting for a minimum of 6 months after surgery. The definition of a complication was based on individual patient reports, provided in a free text format, of any remaining issues 6 months after tonsil surgery. Complications were categorized as follows: disturbed taste or sense of smell, dysphagia, miscellaneous and general symptoms and signs, miscellaneous throat problems, pain or discomfort in the mouth or throat, problems with jaws or teeth, problems with the ears or hearing, problems with the nose or sinuses, problems with throat secretions or throat clearing, problems with voice or speech, and sensory symptoms. A multivariable logistic regression analysis was used to identify independent predictors of long-term complications.

**Results:**

In total, 54,462 patients were included in the study. A total of 3,780 patients (6.9%) reported one or more long-term complications. The most frequent long-term complications, with a plausible connection to the surgery, were found in the following categories: pain or discomfort in the mouth or throat (1.9%), problems with throat secretions or throat clearing (0.8%), dysphagia (0.6%), and problems with voice or speech (0.6%). Tonsillotomy was associated with a lower risk of long-term complications than tonsillectomy.

**Conclusion:**

This study suggests that subjective long-term complications after tonsil surgery, in general, are relatively common (6.9%). However, complications with a plausible connection to the surgery were less common (4.0%), and specific complications seemed to be relatively rare, with no single specific problem reaching a prevalence of ≥0.6%.

## Introduction

Tonsil surgery is one of the most common surgical procedures in otorhinolaryngology. In Sweden, a country with over 10 million inhabitants, approximately 13,500 tonsil surgeries are performed yearly (1). Sleep-disordered breathing and infection-related problems are the two most common indications. In Sweden, both tonsillectomy (TE), complete removal of the tonsils, and tonsillotomy (TT), partial removal of the tonsils, are performed (2). Tonsil surgery is associated with significant postoperative complications and morbidity, with pain and hemorrhage being the most recognized. These complications are well described in the literature and often referred to as early (i.e., within the first weeks) postoperative complications (3–5).

Less is known about problems remaining after the first postoperative period, i.e., long-term complications. Most published reports on the topic have been single-center case series with low numbers of included patients. Taste disorders, velopharyngeal insufficiency, nasopharyngeal stenosis, and calcified stylohyoid ligament (Eagle's syndrome) are some of the long-term complications described in the literature (6, 7). The design of previous studies makes it likely that the true variety and incidence of complications are underestimated. To our knowledge, there are no large population-based studies that have evaluated long-term complications after tonsil surgery.

The Swedish Quality Register for Tonsil Surgery (SQTS) was established in 1997 by the Swedish Association for Otorhinolaryngology, Head and Neck Surgery. Over the last decade, approximately 80% of all tonsil surgeries in Sweden have been included in the register, and by the end of 2020, more than 100,000 surgeries have been registered (8).

Six months after surgery, all patients registered in the SQTS are asked to fill in a questionnaire that focuses on the outcome of surgery including new problems that may have arisen after the operation.

It has previously been stated that well-designed register-based research is well suited to evaluate late and unusual outcomes of surgical interventions (9). This study aimed to describe long-term complications after tonsil surgery that patients report 6 months after surgery. We also aimed to evaluate whether age at surgery, gender, indication for surgery, surgical method, and surgical technique were related to different types of long-term complications.

## Materials and methods

### Study design

This was an exploratory retrospective cohort study based on prospectively collected data from the SQTS.

### Data source and definition of a long-term complication

The SQTS contains individually based information on patients undergoing tonsil surgery for benign causes (i.e., malignant diseases are exclusion criteria for the SQTS). A detailed description of the structure of the register, the included variables and indications, and the data collection procedure is available in English at the SQTS website (8). In connection to the surgery, the surgeon registers a perioperative form with information on age, gender, indication (obstruction or infection), surgical method (TE or TT with or without concomitant adenoid surgery), and surgical technique. The surgical technique was dichotomized in this study as either cold (cold steel instruments used for dissection with cold hemostasis) or hot (electrosurgical instruments used either for dissection or for hemostasis).

Six months after surgery, the patients (or their caregivers) are requested, by mail or email, to complete a questionnaire on the outcome of surgery. One of the questions, “Have you/your child had other problems?” is directly aimed at new problems that may have arisen after the surgery. If the patients have experienced any new problems related to the surgery, the respondent is asked to describe the new problems in a free text format: “If yes, namely…?”. Responses in this free text item were evaluated as potential long-term complications after tonsil surgery in this study.

### Study population

All patients registered in the SQTS between 1 January 2009 and 31 May 2021 and who returned the 6-month follow-up questionnaire were included in the study.

### Data assessment

A stepwise exploratory approach was used to analyze the free text format answers regarding long-term complications.

First, based on the information from previous scientific publications and the authors’ knowledge and experience, a set of main categories with subgroups of long-term complications was defined.

Second, these categories with subgroups were applied to a pilot sample of 1,500 free text format answers. These answers were evaluated by two of the authors (EO and OS). Categories and subgroups that were found to be irrelevant were discarded, and new categories and subgroups were defined as appropriate. Some free text format answers contained irrelevant information, and these were sorted into a separate “Non-complication” category with the following subgroups: “Symptoms not resolved by surgery,” “Postoperative complications that have resolved,” “Complements to staff and/or positive experiences,” and “Commentaries without relevance to the tonsil surgery.” Eleven categories of long-term complications were identified, which in alphabetical order are as follows: “Disturbed taste or sense of smell,” “Dysphagia,” “Miscellaneous and general symptoms and signs,” “Miscellaneous throat problems,” “Pain or discomfort in the mouth or throat,” “Problems with jaws or teeth,” “Problems with the ears or hearing,” “Problems with the nose or sinuses,” “Problems with throat secretions or throat clearing,” “Problems with voice or speech,” and “Sensory symptoms.” The 11 main categories were divided into 63 subcategories.

Third, all long-term complications from the free text format answers were evaluated and categorized into one of the 63 subcategories. To ensure a fair and common assessment, a manual was constructed with examples and explanations on how to categorize. Each free text format answer was assessed and categorized by two separate monitors blinded to each other and to the other variables included in the SQTS. A total of eight monitors, all members of the SQTS board, took part in the evaluation, and each monitor evaluated approximately 1,200 free text format answers. Some patients reported more than one complication. If there was a disagreement between two monitors, the first author (EO) decided the categorization.

### Statistical analysis

The distribution of variables is given as the number and percentage for categorical variables and as the mean, standard deviation (SD), median, minimum, and maximum for continuous variables. For comparisons between groups, Fisher's exact test (lowest one-sided *p*-value multiplied by 2) was used for dichotomous variables, the chi-squared test was used for non-ordered categorical variables, and the *t*-test was used for continuous variables.

TE and TT procedures were analyzed separately with the rationale that TT is associated with a lower risk of short-term postoperative complications compared to TE and that TE is a more extensive surgery with a likely higher risk of harm to underlying or surrounding tissues and structures and thereby also with a higher risk for long-term complications than TT (10).

Uni- and multivariable logistic regression analyses were used to identify predictors of long-term complications in each main category. The variables tested in the univariable logistic regression were age at surgery [odds ratio (OR) per 10 years], sex, indication (obstruction/infection), simultaneous adenoidectomy, and surgical technique (cold/hot). The variables were then entered into a multivariable logistic regression analysis to identify independent predictors. Only main categories with more than 50 events were tested in the multivariable logistic regression analysis. The results from the multivariable logistic regression analyses are given as OR with 95% confidence intervals and *p*-values.

The following categories of complications were excluded from the comparisons between TE and TT procedures and the regression analyses: “Miscellaneous and general symptoms and signs,” “Miscellaneous throat problems,” “Problems with the ears or hearing,” and “Problems with the nose or sinuses.” We chose to exclude these categories from the analysis because we could not identify any reasonable or likely direct connection to the surgery performed. Figures based on all 11 categories were presented as “all reported complications,” and those based on seven categories were considered and reported as “probable complications.”

All significance tests were two-sided and conducted at the 5% significance level. SAS Software Version 9.4 (SAS Institute, NC, USA) was used for all statistical analyses.

## Results

### Participants

Data from a total of 122,308 patients were registered in the SQTS between 1 January 2009 and 31 May 2021. Of these, 54,462 patients or their caregivers returned the 6-month follow-up questionnaire and were included in the study population.

### Descriptive data

Slightly more females than males were included in the study population (51.1% vs. 48.9%), and the mean age at surgery was 13.1 years. The most common indication for surgery was snoring/upper airway obstruction/tonsil hypertrophy (61.7%), followed by recurrent tonsillitis (18.1%). Infection-related indications (recurrent tonsillitis, peritonsillitis, and chronic tonsillitis) accounted for 35.9% of the main indications in the study population. In total, 30,559 patients underwent TE, with or without simultaneous adenoidectomy, and 23,903 patients underwent TT, with or without simultaneous adenoidectomy.

The study population was largely comparable to the non-responders to the 6-month questionnaire. General characteristics of the study population and a comparison with the non-responders to the 6-month questionnaire are given in Table 1.

**Table 1:** General characteristics of the study population and a comparison with the non-responders to the 6-month follow-up questionnaire.

	Study population (*n* = 54,462)	Non-responders (*n* = 67,846)	*p*-value
Sex			
Male	26,608 (48.9%)	34,128 (50.3%)	<0.0001
Female	27,854 (51.1%)	33,718 (49.7%)	
Age at surgery (years)	13.1 (12.9)	12.9 (11.7)	0.0002
	7 (0; 88)	8 (0; 104)	
	(13.0; 13.2)	(12.8; 13.0)	
	*n* = 54,462	*n* = 66,463	
Main indication			
Snoring/upper airway obstruction/tonsil hypertrophy	33,576 (61.7%)	41,414 (61.0%)	<0.0001
Recurrent tonsillitis^a^	9,876 (18.1%)	12,937 (19.1%)	
Peritonsillitis	2,674 (4.9%)	3,309 (4.9%)	
Chronic tonsillitis^b^	7,028 (12.9%)	8,776 (12.9%)	
Systemic complication to tonsillitis	66 (0.1%)	78 (0.1%)	
Other indication	1,242 (2.3%)	1,332 (2.0%)	
Type of surgery			
Tonsillectomy	22,920 (42.1%)	28,149 (42.4%)	<0.0001
Tonsillectomy with adenoidectomy	7,639 (14.0%)	10,579 (15.9%)	
Tonsillotomy	3,444 (6.3%)	3,591 (5.4%)	
Tonsillotomy with adenoidectomy	20,459 (37.6%)	24,144 (36.3%)	
Surgical technique			
Cold	5,768 (10.6%)	8,594 (12.7%)	<0.0001
Hot	47,954 (88.1%)	56,899 (83.9%)	
Unknown	740 (1.4%)	2,353 (3.5%)	

For categorical variables, *n* (%) is presented. For continuous variables, mean (SD)/median (min; max)/(95% confidence interval for mean)/*n* = is presented.

^a^ Recurrent tonsillitis: at least three episodes of tonsillitis in the last year.

^b^ Chronic tonsillitis: at least 3 months with tonsil inflammation that affects daily activities.

### Outcomes

A total of 4,891 patients left a free text format answer in the 6-month questionnaire. Of these, 1,111 answers were categorized into the “Non-complication” category with the following distribution: “Symptoms not resolved by surgery” (*n* = 488), “Commentaries without relevance to the tonsil surgery” (*n* = 456), “Postoperative complications that had resolved” (*n* = 129), and “Complements to staff/positive experience” (*n* = 38).

In total, 3,780 patients (6.9%) reported one or more complications that were categorized into one of the 11 main categories. The numbers and frequencies of all self-reported complications 6 months after surgery, by the 11 main categories with subgroups, are given in Table 2.

**Table 2:** Number and frequency of all self-reported complications.

Main categories of complications with subgroups	Study population (*N* = 54,462)
Pain or discomfort in the mouth or throat^a^	1,057 (1.9%)
Throat pain	331 (0.6%)
Globus or a feeling of a tight throat	288 (0.5%)
Chafing, burning throat, or heartburn	215 (0.4%)
Dry mouth or throat	182 (0.3%)
Gagging	50 (0.1%)
Other symptoms of discomfort in the throat	18 (0.0%)
Tongue pain	6 (0.0%)
Miscellaneous and general symptoms and signs^a^	857 (1.6%)
Upper airway infections	248 (0.5%)
Cough	191 (0.4%)
Breathing problems	68 (0.1%)
Increased frequency of infections and illnesses	63 (0.1%)
Asthma	62 (0.1%)
Pneumonia or bronchitis	50 (0.1%)
Allergy	40 (0.1%)
Stomach problems	37 (0.1%)
Headaches	36 (0.1%)
Miscellaneous and unspecified problems with lymph nodes	29 (0.1%)
Tiredness	22 (0.0%)
Fever	16 (0.0%)
Muscular and/or joint pain	11 (0.0%)
Sleeping problems	7 (0.0%)
Neck pain	5 (0.0%)
Problems with the nose and sinuses^a^	458 (0.8%)
Nasal obstruction	166 (0.3%)
Epistaxis	164 (0.3%)
Coryza	54 (0.1%)
Sinusitis	44 (0.1%)
Nasal pain	27 (0.0%)
Sneezing	9 (0.0%)
Nasal dryness or itching	7 (0.0%)
Problems with sneezing or blowing the nose	3 (0.0%)
Problems with throat secretions or throat clearing^a^	418 (0.8%)
Secretions in the upper airway	302 (0.6%)
Need to clear the throat	122 (0.2%)
Drooling	3 (0.0%)
Problems with voice or speech^a^	344 (0.6%)
Hoarseness	110 (0.2%)
Change of voice	81 (0.1%)
Hyper- or hyponasal speech	71 (0.1%)
Problems with speech articulation	51 (0.1%)
Problems with a rough voice	10 (0.0%)
Problems with singing	10 (0.0%)
Other problems with voice or speech	7 (0.0%)
Tired voice	4 (0.0%)
Pitched voice	2 (0.0%)
Dysphagia^a^	320 (0.6%)
Difficulties to swallow	250 (0.5%)
Regurgitation of food or drink to the nose	75 (0.1%)
Miscellaneous throat problems^a^	287 (0.5%)
Blisters	97 (0.2%)
*Foetor ex ore*	90 (0.2%)
Excavation in tonsil fossa	45 (0.1%)
Deviant appearance in the oropharynx	57 (0.1%)
Disturbed taste or sense of smell^a^	223 (0.4%)
Taste dysfunction—dysgeusia and/or phantogeusia	137 (0.3%)
Taste dysfunction—hypogeusia	45 (0.1%)
Taste dysfunction—ageusia	19 (0.0%)
Taste dysfunction—miscellaneous	17 (0.0%)
Decreased sense of smell	12 (0.0%)
Problems with the ears and hearing^a^	206 (0.4%)
Ear pain	69 (0.1%)
Ear infections	61 (0.1%)
Other or unspecified problems with the ears	35 (0.1%)
Hearing loss, tinnitus, hyperacusis, or dysacusia	30 (0.1%)
Ear fullness, including secretory otitis	24 (0.0%)
Problems with jaws or teeth	44 (0.1%)
Temporomandibular joint dysfunction	31 (0.1%)
Bruxism	9 (0.0%)
Tooth damage	4 (0.0%)
Sensory symptoms	14 (0.0%)
Sensory impairment of the tongue	12 (0.0%)
Other sensory disturbances in the upper airway	2 (0.0%)

For categorical variables, *n* (%) is presented.

^a^ Note that some patients have reported more than one complication in the same category.

Long-term complications for TE and TT procedures are presented in Table 3, where only data from the seven categories with probable complications are included. In total, 2,184 patients (4.0%) reported one or more complications categorized as probable complications. The prevalence of probable long-term complications (one or more) was higher after TE than that after TT (5.8% and 1.8%, respectively).

**Table 3:** Number and frequency of probable self-reported complications by the surgical method.

Subgroup	Total *n*	Pain or discomfort in the mouth or throat	Problems with throat secretions or throat clearing	Dysphagia	Problems with voice or speech	Disturbed taste or sense of smell	Problems with jaws or teeth	Sensory symptoms
Tonsillectomy	30,559	908 (3.0%)	305 (1.0%)	276 (0.9%)	225 (0.7%)	219 (0.7%)	36 (0.1%)	14 (0.0%)
Age (years)								
0–17	15,668	182 (1.2%)	82 (0.5%)	43 (0.3%)	92 (0.6%)	14 (0.1%)	20 (0.1%)	1 (0.0%)
18+	14,891	726 (4.9%)	223 (1.5%)	233 (1.6%)	133 (0.9%)	205 (1.4%)	16 (0.1%)	13 (0.1%)
Sex								
Male	13,086	255 (1.9%)	113 (0.9%)	72 (0.6%)	78 (0.6%)	76 (0.6%)	9 (0.1%)	4 (0.0%)
Female	17,473	653 (3.7%)	192 (1.1%)	204 (1.2%)	147 (0.8%)	143 (0.8%)	27 (0.2%)	10 (0.1%)
Indication								
Infection	19,302	677 (3.5%)	212 (1.1%)	212 (1.1%)	132 (0.7%)	170 (0.9%)	26 (0.1%)	11 (0.1%)
Obstruction	10,201	190 (1.9%)	77 (0.8%)	50 (0.5%)	88 (0.9%)	31 (0.3%)	9 (0.1%)	1 (0.0%)
Adenoidectomy								
Yes	7,639	56 (0.7%)	46 (0.6%)	24 (0.3%)	58 (0.8%)	7 (0.1%)	2 (0.0%)	0 (0.0%)
No	22,920	852 (3.7%)	259 (1.1%)	252 (1.1%)	167 (0.7%)	212 (0.9%)	34 (0.1%)	14 (0.1%)
Surgical technique								
Cold	5,193	145 (2.8%)	40 (0.8%)	25 (0.5%)	47 (0.9%)	20 (0.4%)	3 (0.1%)	2 (0.0%)
Hot	24,867	754 (3.0%)	260 (1.0%)	240 (1.0%)	177 (0.7%)	193 (0.8%)	32 (0.1%)	11 (0.0%)
Unknown	499	9 (1.8%)	5 (1.0%)	11 (2.2%)	1 (0.2%)	6 (1.2%)	1 (0.2%)	1 (0.2%)
Tonsillotomy	23,903	149 (0.6%)	113 (0.5%)	44 (0.2%)	119 (0.5%)	4 (0.0%)	8 (0.0%)	0 (0.0%)
Age (years)								
0–17	23,233	126 (0.5%)	109 (0.5%)	39 (0.2%)	117 (0.5%)	3 (0.0%)	8 (0.0%)	0 (0.0%)
18+	670	23 (3.4%)	4 (0.6%)	5 (0.7%)	2 (0.3%)	1 (0.1%)	0 (0.0%)	0 (0.0%)
Sex								
Male	13,522	72 (0.5%)	68 (0.5%)	23 (0.2%)	68 (0.5%)	2 (0.0%)	4 (0.0%)	0 (0.0%)
Female	10,381	77 (0.7%)	45 (0.4%)	21 (0.2%)	51 (0.5%)	2 (0.0%)	4 (0.0%)	0 (0.0%)
Indication								
Infection	342	8 (2.3%)	0 (0.0%)	2 (0.6%)	0 (0.0%)	0 (0.0%)	0 (0.0%)	0 (0.0%)
Obstruction	23,375	136 (0.6%)	112 (0.5%)	42 (0.2%)	118 (0.5%)	4 (0.0%)	8 (0.0%)	0 (0.0%)
Adenoidectomy								
Yes	20,459	106 (0.5%)	92 (0.4%)	33 (0.2%)	105 (0.5%)	4 (0.0%)	8 (0.0%)	0 (0.0%)
No	3,444	43 (1.2%)	21 (0.6%)	11 (0.3%)	14 (0.4%)	0 (0.0%)	0 (0.0%)	0 (0.0%)
Surgical technique								
Cold	575	3 (0.5%)	2 (0.3%)	2 (0.3%)	2 (0.3%)	0 (0.0%)	0 (0.0%)	0 (0.0%)
Hot	23,087	143 (0.6%)	111 (0.5%)	41 (0.2%)	115 (0.5%)	4 (0.0%)	8 (0.0%)	0 (0.0%)
Unknown	241	3 (1.2%)	0 (0.0%)	1 (0.4%)	2 (0.8%)	0 (0.0%)	0 (0.0%)	0 (0.0%)

For categorical variables, *n* (%) is presented.

#### Pain or discomfort in the mouth or throat

**Figure 1:**
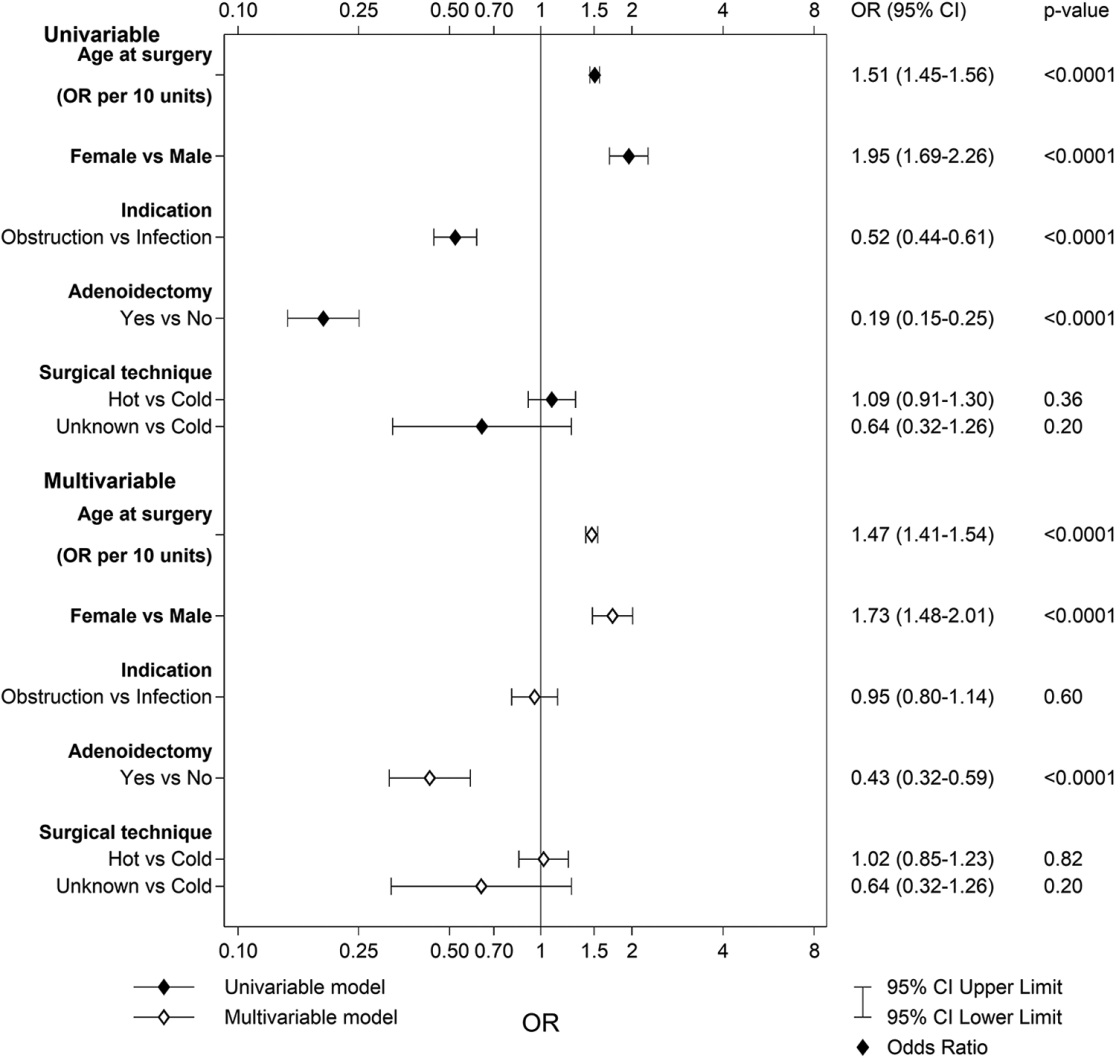
Univariable and multivariable predictors of pain or discomfort in the mouth or throat after tonsillectomy.

A total of 908 patients (3.0%) in the TE group and 149 patients (0.6%) in the TT group reported pain or discomfort in the mouth or throat 6 months after surgery. As seen in Figure 1, older age at surgery, female sex, and no simultaneous adenoidectomy increased the risk for pain or discomfort in the mouth and/or throat after a TE. The area under the ROC (receiver operating characteristic) curve with 95% confidence interval (CI) for the multivariable model for TE was 0.72 (0.71–0.74). In the TT group, only older age at surgery was indicated to be an individual risk factor for this complication [*p* < 0.0001; OR 1.79 (95% CI 1.53–2.10)]. The area under the ROC curve with 95% CI for the multivariable model for TT was 0.65 (0.60–0.70).

#### Problems with throat secretions or throat clearing

**Figure 2:**
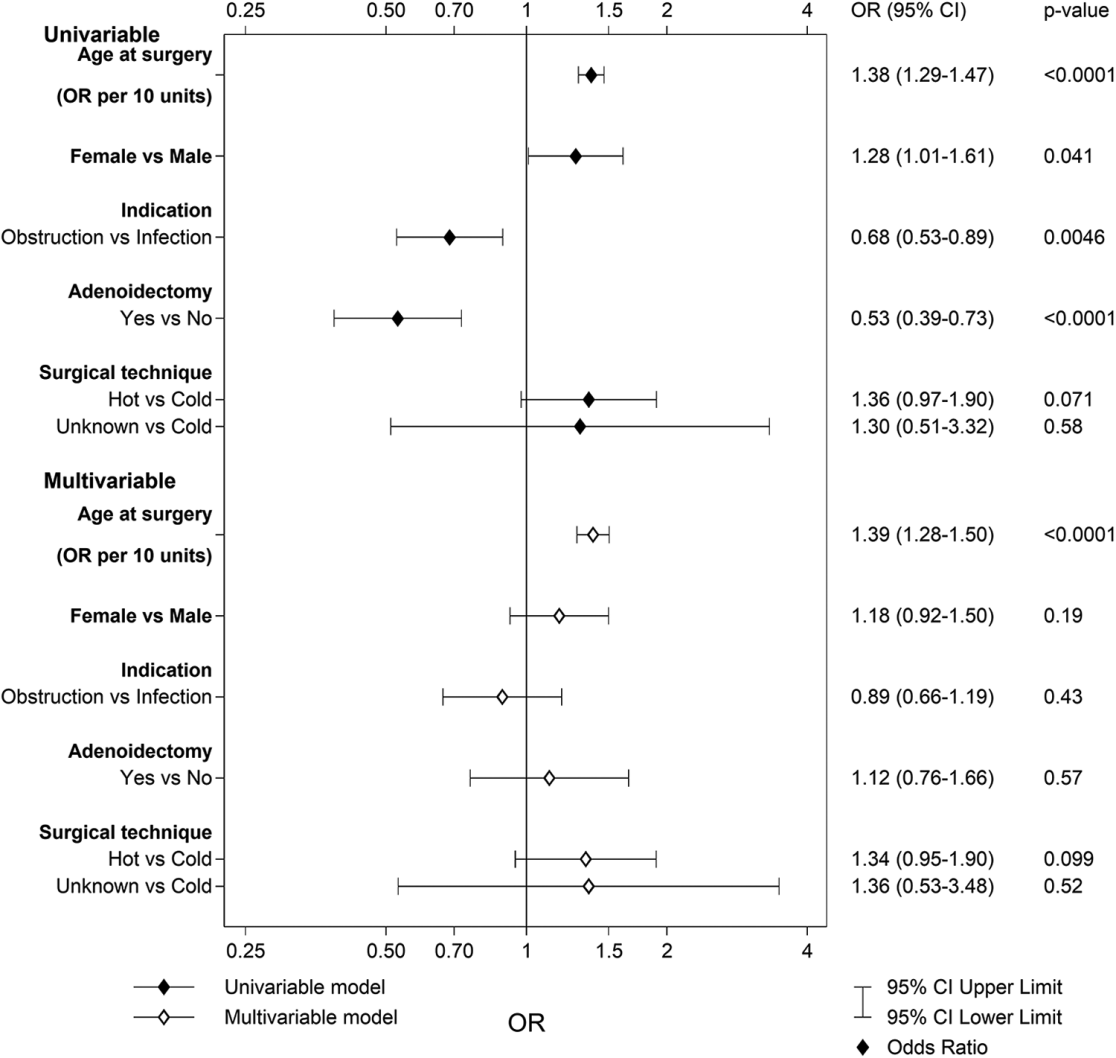
Univariable and multivariable predictors of problems with throat secretions or throat clearing after tonsillectomy.

A total of 305 (1.0%) patients in the TE group and 113 (0.5%) in the TT group reported problems with throat secretions and/or throat clearing. The multivariable logistic regression for the TE group revealed that older age at surgery was the only individual risk factor for this complication (Figure 2). The area under the ROC curve with 95% CI for the multivariable model for TE was 0.66 (0.63–0.69). The multivariable logistic regression for the TT group revealed no statistically significant risk factors: age at surgery [*p* = 0.054; OR 1.33 (95% CI 0.99–1.76)], sex [*p* = 0.31; OR 0.82 (95% CI 0.56–1.20)], indication [*p* = 0.99; OR 1.49 × 10^6^ (95% CI 0.00–Infinity)], adenoidectomy [*p* = 0.37; OR 0.79 (95% CI 0.47–1.33)], and surgical technique [*p* = 0.71; OR 1.31 (95% CI 0.32–5.33)]. The area under the ROC curve with 95% CI for the multivariable model for TT was 0.59 (0.54–0.64).

#### Dysphagia

**Figure 3:**
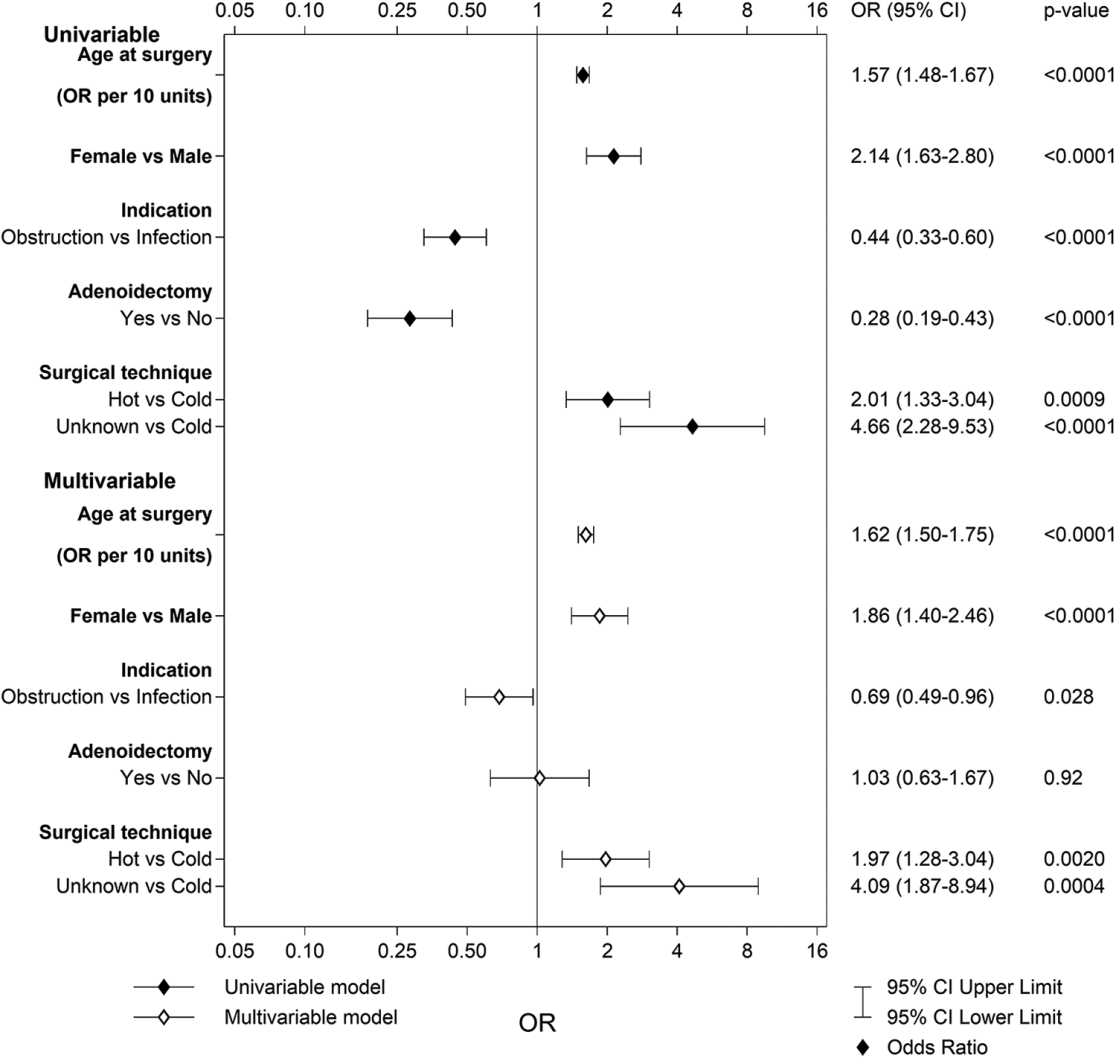
Univariable and multivariable predictors of dysphagia after tonsillectomy.

A total of 276 patients (0.9%) in the TE group and 44 patients (0.2%) in the TT group reported dysphagia (difficulties swallowing and/or regurgitation of food or drink to the nose) 6 months after surgery. The multivariable logistic regression analysis for the TE group revealed that older age at surgery, female sex, and hot surgical technique increased the risk for dysphagia (Figure 3). The area under the ROC curve with 95% CI for the multivariable model was 0.76 (0.73–0.78). No multivariable logistic regression analysis was performed for TT due to too few events.

#### Problems with voice or speech

**Figure 4:**
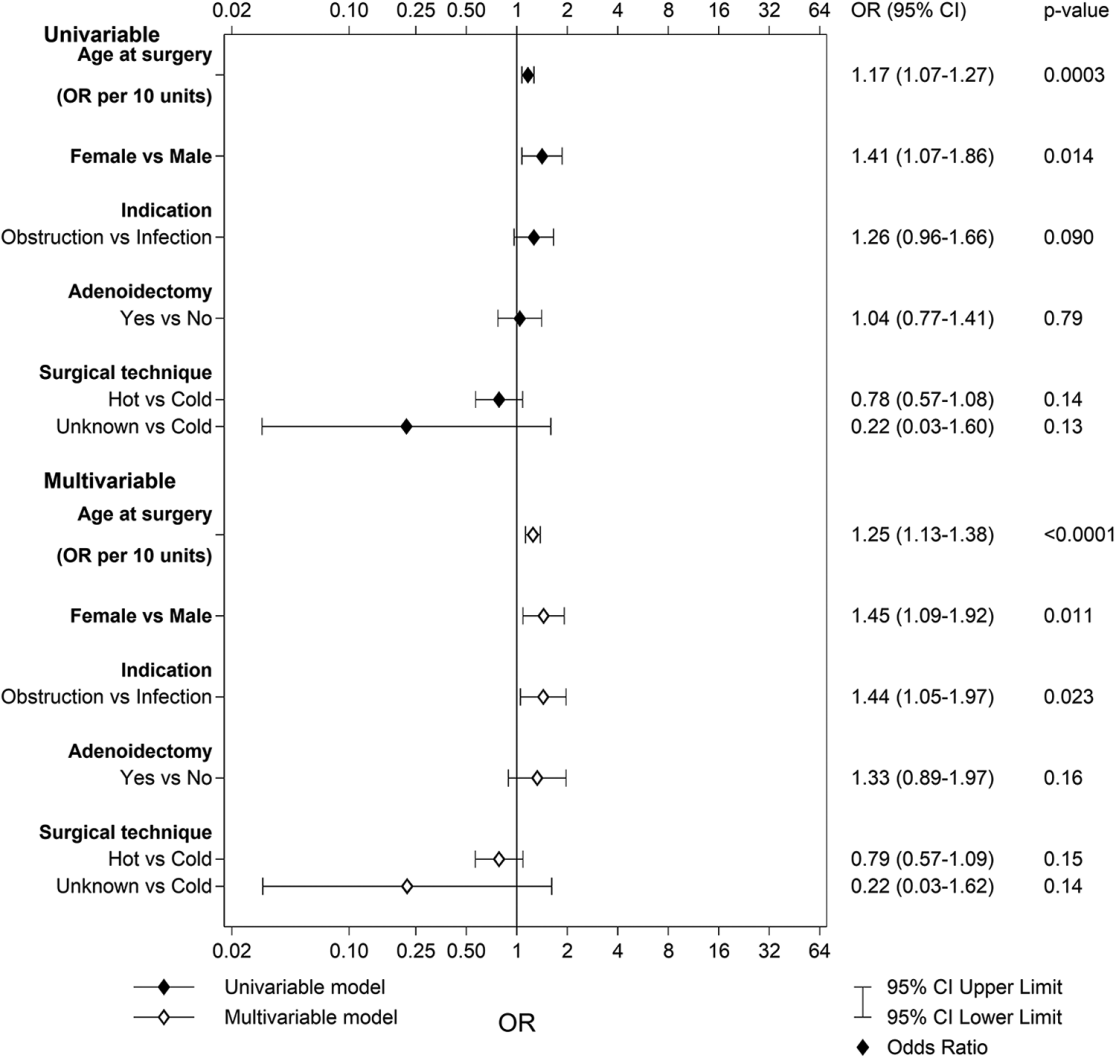
Univariable and multivariable predictors of problems with voice and speech after tonsillectomy.

In the TE group, 225 patients (0.7%) compared to 119 (0.5%) in the TT group reported problems with voice or speech 6 months after surgery. The multivariable logistic regression analysis for the TE group revealed that older age at surgery, female sex, and infection as indication for surgery led to an increased risk for problems with voice or speech (Figure 4). The area under the ROC curve with 95% CI for the multivariable model for TE was 0.61 (0.57–0.65). The multivariable logistic regression for the TT group revealed no statistically significant risk factors: age at surgery [*p* = 0.18; OR 0.67 (95% CI 0.38–1.20)], sex [*p* = 0.89; OR 1.03 (95% CI 0.71–1.48)], indication [*p* = 0.98; OR 301,165 (95% CI 0.00–Infinity)], adenoidectomy [*p* = 0.97; OR 1.01 (95% CI 0.57–1.81)], and surgical technique [*p* = 0.64; OR 1.40 (95% CI 0.35–5.69)]. The area under the ROC curve with 95% CI for the multivariable model for TT was 0.56 (0.51–0.61).

#### Disturbed taste or sense of smell

**Figure 5:**
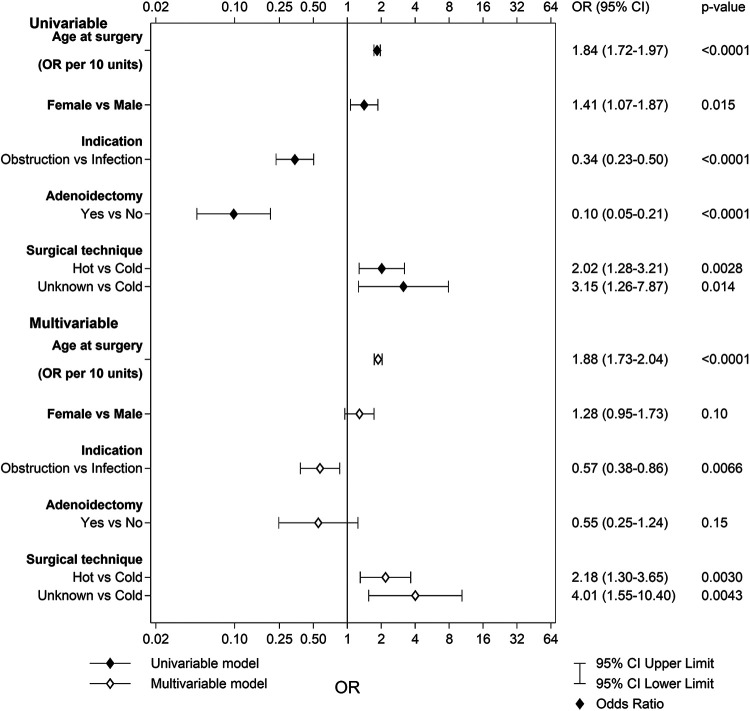
Univariable and multivariable predictors of disturbed taste or sense of smell after tonsillectomy.

In the TE group, 219 patients (0.7%) reported disturbed taste or sense of smell 6 months after surgery compared to 4 patients (0.0%) in the TT group. The multivariable logistic regression analysis for TE indicated that older age at surgery and using a hot surgical technique led to an increased risk of disturbed taste or sense of smell, whereas obstruction as a main indication decreased the risk (Figure 5). The area under the ROC curve with 95% CI for the multivariable model was 0.84 (0.81–0.86). No multivariable logistic regression analysis was performed for TT due to too few events.

#### Problems with jaws or teeth

A total of 36 patients (0.1%) in the TE group and eight patients (0.0%) in the TT group reported problems with jaws or teeth 6 months after surgery. No multivariable logistic regression analyses were performed for either TE or TT due to too few events.

#### Sensory symptoms

Sensory symptoms including “sensory impairment of the tongue” and “other sensory disturbances in the upper airway” were rare complications, with only 14 (0.0%) patients in the TE group and no patients in the TT group reporting these complications. No multivariable logistic regression analyses were made for either group due to too few events.

## Discussion

In this study, we evaluated long-term complications reported by patients 6 months after tonsil surgery. To our knowledge, this is the largest study on the topic so far, with over 50,000 patients included. The most difficult part of this study, and its greatest limitation, was the definition of main categories with subgroups of complications and the categorization of thousands of free text format answers. On the other hand, while the free text format caused methodological and interpretational challenges, it also provided unique insight into unfiltered patient experiences. By not using fixed and predetermined response options, we had the opportunity to find and describe the full extent and variety of patient-perceived long-term complications after tonsil surgery. Hence, the most important finding of this study was the recognition of the enormous diversity of patient-perceived problems after tonsil surgery. Many of the problems perceived by the patients may to the clinician seem to have a far-fetched or even an impossible connection to the tonsil surgery, while other problems observed in this study may have implications on clinical practice including the preoperative information given to the patients on possible harmful outcomes of surgery.

Are long-term problems after tonsil surgery common? Although 6.9% of the included patients reported one or more problems 6 months after tonsil surgery, the prevalence of probable complications was 4.0% (i.e., after excluding categories of complications where we failed to identify a plausible causal relationship). The prevalence of each individual problem was very low (≤0.6%). As expected, TT was associated with a lower prevalence of long-term complications than TE. This is a logical result as TT is a less extensive surgery than TE, only involving the lymphoid tissue of the tonsils and not affecting underlying and surrounding tissues and structures.

An interesting finding was that a hot surgical technique was an independent risk factor for “Disturbed taste or sense of smell” and “Dysphagia” after TE. The finding is also logical, considering that previous studies have shown that hot (electrosurgical) surgical techniques increase the risk of postoperative hemorrhage after tonsil surgery, probably for the same reasons (11, 12).

Our study is not the first to identify taste disorders as a long-term complication following tonsil surgery (13, 14). In 2018, Soldatova and Doty published a systematic review on post-TE taste dysfunction (15). In this review, including eight studies, almost all cases of taste disorders resolved within 6 months of surgery. The authors concluded that “the available literature does not provide enough information to estimate the prevalence, duration and nature of post-TE taste disorders” (15). Direct or indirect injury to the lingual or tonsillar branches or the glossopharyngeal nerve from ligation or stretching of the nerve or from scarring of the nerve during the healing period is the potential cause of postoperative taste disorders (15). The use of hot techniques has already been proposed as a risk factor for post-TE taste disorders, and minimal use of these instruments has been recommended (13). In contrast, later studies have shown no association with using hot techniques to achieve hemostasis and postoperative taste disorders (14). In our study, a hot surgical technique increased the risk of disturbed taste or sense of smell after TE with OR of 2.18 compared to a cold technique. Thus, even though taste disorders and dysphagia seem to be uncommon long-term problems after tonsil surgery, they may be devastating to the few who are affected. The risk for taste disorders related to hot surgical techniques is another reason, in addition to the increased risk for postoperative hemorrhage (11), why cold techniques should be used in TE.

Older age at surgery has previously been described as a risk factor for short-term complications especially when focusing on postoperative hemorrhage and pain (16–18). The reason for this is not fully understood, and older age also seems to be an individual risk factor in studies using multivariable regression models (19–21). In our study, older age at surgery was an independent risk factor for the following categories: “Disturbed taste or sense of smell,” “Pain or discomfort in the mouth or throat,” “Dysphagia”, “Problems with voice or speech,” and “Problems with secretions or throat clearing.” There are some hypothetical explanations. Children may be both less prone to identify these problems (and tell the caregivers who were the respondents in this study) and more prone to adapt to a postoperative change in the throat. Another explanation is that the often longstanding tonsil disease in adults has involved surrounding tissues to a higher extent compared to children, making the needed surgery more extensive and thereby more harmful. Why the female sex was an independent risk factor for five main categories following TE and for one of seven main categories after TT remains poorly understood and stands in contrast to previous findings where female sex led to a decreased risk for early postoperative morbidity such as hemorrhage, analgesic use, and antibiotic use (19).

Infection-related indications were a significant risk factor for “Disturbed taste or sense of smell” after TE and for “Dysphagia” after TE. Infection-related indications have previously been proposed as a risk factor for short-term complications, e.g., postoperative hemorrhage and pain (19, 21), while other studies have failed to establish such a relationship (22). The most likely explanation of our finding is that infectious tonsil disease, in most cases, is a chronic disease involving surrounding tissue, especially the tonsil fossae, to a higher degree than hypertrophic disease. Hypothetically, the surgery has to be more extensive and, thereby, more harmful.

Obstruction-related indications, female sex, and older age were individual risk factors for the “Problems with voice or speech” category after TE. Interestingly, a simultaneous adenoidectomy was not a risk factor. The most common complaints in this category were hoarseness, change of voice, and hyper- or hyponasal speech. An explanation for this could be the increase of space in the oropharynx following a TE with an obstruction-related indication, leading to a change in resonance. Why older and female patients experienced (or reported) these problems to a higher extent than younger or male participants is unclear but may hypothetically be related to psychological or awareness factors.

The finding that a simultaneous adenoidectomy reduced the risk for “Pain or discomfort in the mouth or throat” after TE but not after TT was an unexpected result and cannot easily be explained. A simultaneous adenoidectomy means an addition of a surgical field and would intuitively lead to an increased risk of complications. In a previous study comparing short-term complications after TE vs. TT, the same and, so far, unexplained finding was observed (10).

A total of 857 patients (1.6%) reported symptoms 6 months after surgery that were categorized as “Miscellaneous and general symptoms and signs.” This was a heterogeneous category of complications with the top five problems: “Upper airway infections” (*n* = 248, 0.5%), “Cough” (*n* = 191, 0.4%), “Breathing problems” (*n* = 68, 0.1%), “Increased frequency of infections” (*n* = 63, 0.1%), and “Asthma” (*n* = 62, 0.1%). In 2018, Byars et al. published a population-based cohort study, including almost 1.2 million Danish children, on long-term disease risks associated with adenoid and tonsil surgery in childhood (23). The authors concluded that adenoid and tonsil surgery were associated with increased long-term risks of respiratory, infectious, and allergic diseases, but the study faced criticisms mainly due to lack of causality and inherent biases such as confounding, reverse causation, and selection bias (24–26). The number and frequency of these problems were low in our population and may reflect the natural incidence, unrelated to tonsil surgery, of these problems in the general population.

### Methodological considerations

Even though randomized controlled trials are generally viewed as the gold standard for evaluating efficacy and safety for surgical methods, registry-based studies are promoted for assessing long-term effects and complications (27). The strengths of a well-designed registry-based study lie in recognizing late or uncommon outcomes. Furthermore, registries allow evaluation of real-world outcomes in an uncontrolled environment.

This study had some limitations. The long-term complications were retrieved through a questionnaire 6 months after surgery, where the respondents were asked to describe any new problems following the surgery in a free text format. The free text format answers were evaluated and categorized into one of 63 subcategories by the authors. There was a risk of bias in the process of analyzing and categorizing the free text format answers. To minimize the risk of inadequate evaluation, every free text format answer was separately assessed and categorized by two monitors who were blinded to each other.

Given the nature of information retrieval, we lack information on the severity of the reported complication. Another limitation is that we lack access to medical records and therefore no access to standardized medical evaluations for some of the reported complications, e.g., asthma, disturbed taste, and others.

A potential limitation and source of selection bias is the high number of non-responders (50.3%) to the SQTS 6-month questionnaire. As shown in Table 1, the difference regarding the main characteristics of responders and non-responders are small but statistically significant, which must be kept in mind when interpreting the results of this study.

## Conclusion

This study suggests that patient-reported subjective problems related to a tonsil surgery performed 6 months prior, in general, were relatively common (6.9%). However, the prevalence of complications with a likely causal relationship with the surgery was not that common (4.0%), and each specific complication seemed to be rather rare, with no single reported complication reaching a prevalence of ≥0.6%. A main finding is the diversity and quantity of problems reported by the patients, with many reported problems having a far-fetched or clinically doubtful connection to the tonsil surgery. Nevertheless, some of the reported problems must be considered long-term complications to tonsil surgery and should influence clinical practice, for example, surgical method and surgical technique. The results of this study should also influence the preoperative information given to the patients about the potential long-term risks of tonsil surgery so that informed decisions can be made.

## Data Availability

The datasets presented in this article are not readily available because data from the Swedish Quality Register for Tonsil Surgery (SQTS) is regulated by Swedish law. Research using SQTS data may be carried out following approval by an ethics committee. Please contact the Centre of Registers Västra Götaland in Sweden for more information. Requests to access the datasets should be directed to registercentrum@vgregion.se.
